# The expressed genes of Japanese red pine (*Pinus densiflora*) involved in the pine wilt disease severity

**DOI:** 10.1186/1753-6561-5-S7-P92

**Published:** 2011-09-13

**Authors:** Hiroyuki Kuroda, Susumu Goto, Etoh Kazumi, Keiko Kuroda

**Affiliations:** 1 Institute of Sustainable Science, Kyoto University, Uji, Kyoto, Japan; 2 Institute for Chemical Research, Kyoto University, Uji, Kyoto, Japan; 3 Wood Research Institute, Kyoto University, Uji, Kyoto, Japan; 4 Graduate School of Agricultural Science, Kobe University, Kobe, Japan

## Background

Japanese pine trees have been severely damaged by the pine wilt diseases, and hardly been keeping the productive forest in Japan. The pine wood nematode,*Bursaphelenchus xylophilus,* is a virulent pathogen in Japan, while it is originated and is an endemic one in the United State [1]. The diseases have now been spread not only in East Asia but also in a part of Europe. The Japanese Government has been developed the resistant pine breed varieties which had selected the survived-tree lineages obtained from the damaged pine forests. The developed varieties vary in the disease severity depending on the individual trees even in the same variety, partly because of the wind-pollination. Thus it needs molecular indices to select the resistant individuals without nematode infection.

Shin et al.[2] reported up-regulated genes in a non-selected, or susceptible, Japanese red pine, *Pinus densiflora*, after the nematode inoculation . Defense responses distinct from the susceptible trees may be expressed in the resistant bleed variety against the pine wood nematodes. By comparing the expressed gene profiles between the resistant and susceptible varieties against the diseases, a molecular clue for the resistant response against the diseases will be provided. Here, we have compared gene expression profiles between the susceptible and resistant breed varieties of Japanese red pine at the stage when the defense response had just established after the nematode inoculation. We also discuss on the gene components involved in the disease severity or resistance.

## Methods

Pine wood nematodes were inoculated on 2-year-old seedlings of a non-selected susceptible and one of the strongest resistant breed variety, respectively. The five samples were harvested at every 7 days and counted the nematode numbers of the samples. They are also stored at -80°C for RNA extraction. The nematodes were collected by Baermann funnel technique and counted under a stereomicroscope to judge the resistance. RNAs were extracted from the resistant and susceptible stems judged at the stage when the nematodes had just started to propagate, by Quagen RNeasy Plant Mini Kit with minor modification. The cDNAs obtained from the resistant and susceptible breed varieties, were differentially screened by Megasort beads technology. The 1507 and 1329 cDNAs, or ESTs, from the respective breed varieties, were effectively sequenced and assembled as the contigs. They were subjected to Blast retrieval against various public databases for their assignments. The data sets obtained were analyzed *in silico.*

## Results and conclusions

**Figure 1:**
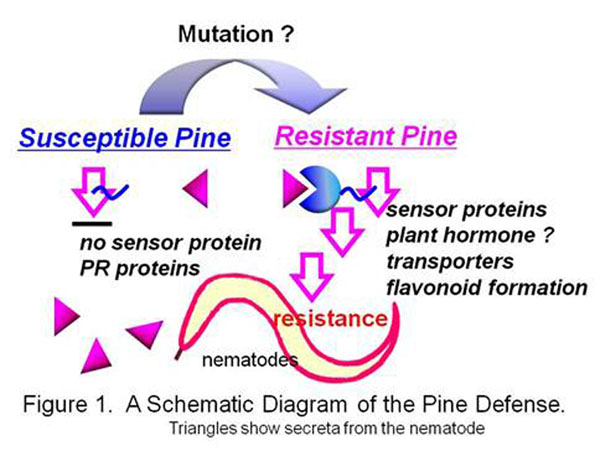
A schematic diagram of the pine defense. Triangles show secreta from the nematode.

A contig sequence is composed of ESTs, and it approximates an expressed gene species, while the numbers of ESTs in a contig approximate the expressed level of the gene. Comprehensive expression analyses show that the contig numbers, i.e. gene species, were more abundant in a resistant breed variety than those in a susceptible one. The contigs expressed more than 4 ESTs, being composed of 803 ESTs, were three times abundant in the resistant variety than in the susceptible one. The defense response might be triggered by the nematode invasion in a resistant variety but not in a susceptible one. Alternatively, it may be caused by the suppressed gene expression in the susceptible one. The latter is more probable because the signal transduction involved in the resistant response had blocked in the susceptible variety (Figure 1).

The flavonoid biosynthesis had been transcriptionally more expressed in the resistant variety than in the case of the susceptible one. One of the authors had suspected that nematicidal stilbenoids, which are abundantly localized in pine heartwood, might be involved in the disease resistance [3]. The stilbenoid biosynthesis was activated by methyljasmonate or salicylate application but did not detect in this experiment at the stage when the resistant variety had just established defending response. The phenolic metabolites were more produced in the resistant variety comparing to the case of the susceptible one, supporting the results from the transcriptional observation. Some pathogen related (PR) proteins were not observed in the resistant breed but were up-regulated in the susceptible one, suggesting such proteins may be induced by stress and not directly involved in the defense response. A cluster, being composed of the contigs, approximates a gene family and represents a hot spot in the mutation which may or may not be involved in the defense response. We will also discuss those gene families.
